# Vitamin C Fosters the *In Vivo* Differentiation of Peripheral CD4^+^ Foxp3^−^ T Cells into CD4^+^ Foxp3^+^ Regulatory T Cells but Impairs Their Ability to Prolong Skin Allograft Survival

**DOI:** 10.3389/fimmu.2018.00112

**Published:** 2018-02-09

**Authors:** Karina Oyarce, Mauricio Campos-Mora, Tania Gajardo-Carrasco, Karina Pino-Lagos

**Affiliations:** ^1^Centro de Investigación Biomédica, Facultad de Medicina, Universidad de los Andes, Santiago, Chile

**Keywords:** vitamin C, regulatory T cells, tolerance, transplantation, Foxp3

## Abstract

Regulatory T cells (Tregs) are critical players of immunological tolerance due to their ability to suppress effector T cell function thereby preventing transplant rejection and autoimmune diseases. During allograft transplantation, increases of both Treg expansion and generation, as well as their stable function, are needed to ensure allograft acceptance; thus, efforts have been made to discover new molecules that enhance Treg-mediated tolerance and to uncover their mechanisms. Recently, vitamin C (VitC), known to regulate T cell maturation and dendritic cell-mediated T cell polarization, has gained attention as a relevant epigenetic remodeler able to enhance and stabilize the expression of the Treg master regulator gene Foxp3, positively affecting the generation of induced Tregs (iTregs). In this study, we measured VitC transporter (SVCT2) expression in different immune cell populations, finding Tregs as one of the cell subset with the highest levels of SVCT2 expression. Unexpectedly, we found that VitC treatment reduces the ability of natural Tregs to suppress effector T cell proliferation *in vitro*, while having an enhancer effect on TGFβ-induced Foxp3^+^ Tregs. On the other hand, VitC increases iTregs generation *in vitro* and *in vivo*, however, no allograft tolerance was achieved in animals orally treated with VitC. Lastly, Tregs isolated from the draining lymph nodes of VitC-treated and transplanted mice also showed impaired suppression capacity *ex vivo*. Our results indicate that VitC promotes the generation and expansion of Tregs, without exhibiting CD4^+^ T cell-mediated allograft tolerance. These observations highlight the relevance of the nutritional status of patients when immune regulation is needed.

## Introduction

Regulatory T cells (Tregs) are a specialized CD4^+^ T cell population that inhibits the proliferation and function of effector T cells, thus enabling immune tolerance (1). They can be divided into two subpopulations based on their origin; natural Tregs (nTregs) that arise from immature naive CD4^+^ T cells in the thymus, and adaptive or induced Tregs (iTregs) that originate in the periphery from mature T cell populations. Both Treg subsets play a crucial role under homeostatic and pathological settings, avoiding autoimmune diseases (2, 3), controlling inflammation (4), increasing tolerance to allotransplanted organs (5, 6), and promoting tumor progression by blocking antitumor effector T cell functions (7, 8). One of the hallmarks of the Treg phenotype is the expression of Foxp3, a transcription factor considered a master regulator of Treg development and function (9, 10). In this regard, loss of Foxp3 expression under *in vivo* or *in vitro* proinflammatory conditions has been related to a deficiency in suppressive capacity and gain of effector T cell functions (becoming pathogenic), which infers the importance of maintaining Treg stability (11–13). Molecular pathways found to promote Treg stability include: (i) Foxp3 stabilization through STAT5 binding to the Foxp3 promoter region, (ii) modulation of IL-2 signaling (14), (iii) triggering of Neuropilin/Semaphorin4a axis signaling (15), (iv) epigenetic remodeling of transcription sites by histone H3-H4 di and trimethylation, and (v) DNA methylation at enhancer regulatory elements sites (16–18).

Because Tregs are considered key players in driving immune tolerance, there is an increased necessity in finding new molecules that can enhance their generation, expansion and suppressive function in a relevant clinical setting. In this context, vitamins play an essential role in physiologic processes, including immune responses (19–23). As an example, vitamins A and D have been extensively studied in immunity, where they regulate inflammatory and anti-inflammatory responses, as reported in several publications using animal models (24–27). Interestingly, vitamins contribute to the *in vitro* and *in vivo* generation of Tregs (26, 28). Vitamin C (VitC), a well-studied micronutrient implicated in cell proliferation, differentiation and maturation processes has also been related to the regulation of the immune system (29, 30). For example, it has been shown that VitC participates in T cell maturation (29), and most of the *in vitro* studies indicate that VitC may act on T cells and dendritic cells (DCs), favoring the development of Th1-type responses over Th2 (31), reducing inflammation in some animal models (32, 33) and promoting the generation of tolerogenic human DCs (34). Most recently, an interesting observation reported the connection between VitC and Tregs, in which the presence of VitC during *in vitro* Tregs induction from naive CD4^+^ T cells could upregulate the expression of Foxp3 (35, 36) and the generation of iTreg (35–37). Furthermore, a new study has revealed that iTreg induced in the presence of both vitamin A and C allows for skin-allograft transplantation tolerance when adoptively transferred (37), however, the effect that VitC alone can have upon Treg function *in vivo* has not yet been tested.

Vitamin C enters the cell in its physiological form through the sodium-dependent VitC transporter 2 (SVCT2), whose expression has been determined in DCs, macrophages, and circulating T cells in general (38–40), however so far, its presence on Treg has not been determined. We analyzed the expression of SVCT2 on different leukocyte populations, and results showed Treg as the cell population expressing the highest levels of SVCT2 mRNA. In addition, we demonstrate that nTregs treated with VitC poorly suppress the proliferation of polyclonally activated CD4^+^ T cells *in vitro*. Conversely, we corroborate that VitC is able to trigger Foxp3 expression on CD4^+^ Foxp3^−^ T cells in a TGF-β-dependent fashion. Interestingly, VitC impacts both the *de novo* expression of Foxp3 and the level of Foxp3 (on already Foxp3^+^ T cells) *in vitro* and *in vivo*, however, no improvement on graft survival is achieved.

Altogether, our data support *in vitro* studies on the role of VitC as an enhancer of Foxp3 expression on Treg, and indicates that regardless of their enhanced Foxp3 expression, VitC-treated Tregs display deficient regulatory function as seen in *in vitro* and *ex vivo* approximations.

## Materials and Methods

### Mice

Six- to eight-week-old C57BL/6 wild-type, C57BL/6 x BALB/c (F1), Foxp3/GFP and RAG-KO mice were used (both under C57BL/6 background). All mice were maintained under pathogen free conditions, with a 12 h light/dark cycle and food and water *ad libitum*. This study was carried out after the revision and approval of its experimental protocol in accordance with the recommendations of bioethical Committee guidelines from the Faculty of Medicine, Universidad de los Andes, and the Comisión Nacional de Ciencia y Tecnología, CONICYT.

### Skin Transplantation

For skin transplantation experiments, mice were anesthetized with a mixture of ketamine at 27% and xylazine at 3% *via* intraperitoneal (i.p.) injection. Tail skin (~1 cm^2^) from C57BL/6 (syngeneic) or F1 (allogeneic) donors was transplanted onto the dorsal area of C57BL/6 or RAG^−/−^ recipient mice. l-Ascorbic acid (AA, Sigma-Aldrich, MI, USA) was added daily in the water at 0.86 mg/mL, starting the day of surgery until the end of the experiment. Survival of skin allografts was evaluated twice per week and grafts were considered rejected when 80% of the original graft had disappeared or become necrotic.

### DCs, B, and T Cell Isolation by Magnetic Separation and Cell Sorting

Dendritic cells were purified from wild-type C57BL/6 mouse spleen, digested in the presence of 1 mg/mL of collagenase D (Roche, Germany) and 2 µg/mL of DNAse I (Roche, Germany) in RPMI medium (Thermo Scientific, NH, USA) supplemented with 10% FBS (Gibco, USA), 1% penicillin/streptomycin (Corning, USA), HEPES (Gibco, Great Britain), β-mercaptoethanol (Sigma, MO, USA) at 50 µM. The 1 mL of this mixture was used to perfuse the organ, followed by an incubation of 45 min at 37°C in waterbath. Undigested material was filtered through a cell strainer (Fisher Scientific, NH, USA) and CD11c^+^ cells were isolated from the obtained cell suspension by using mouse CD11c MicroBeads UltraPure (MACS, Miltenyi, Germany), according to the manufacture instructions. For lymphocytes, spleens from Foxp3/GFP mice were collected in PBS 1× + 5% of FBS and mechanically disaggregated through a cell strainer. Red blood cells were lysed using RBC lysis buffer 10× (Biolegend, CA, USA) and total CD4^+^ T cells were purified using mouse CD4^+^ T cell isolation kit (MACS Miltenyi Biotec, Germany), according to the manufacture instructions. B cells and CD8^+^ T cells were sorted from the CD4^−^ fraction based on the expression of CD8 and CD19, while CD4^+^ T conventional and CD4^+^ T regulatory (Tregs) cells were sorted from the CD4^+^ fraction based on the expression of CD4 and Foxp3/GFP using a BD FACSAria III (Franklin Lakes, NJ, USA).

### Induction of Foxp3^+^ Treg Cells

Total CD4^+^ T cells were isolated from the spleens of Foxp3/GFP mice, using mouse CD4^+^ T cell isolation kit (MACS Miltenyi Biotec, Germany), according to the manufacture instructions. 2 × 10^5^ cells were cultured in 24-well plates, previously coated with anti-CD3 (clone 2c11, at 10 µg/mL) and anti-CD28 (clone PV-1, at 1 µg/mL) for 6 days. Culture media contained RPMI medium (Thermo Scientific, NH, USA) supplemented with 5% FBS (Gibco, USA), HEPES (Gibco, Great Britain), 1% penicillin/streptomycin, and β-mercaptoethanol 50 µM, in the presence of IL-2 (at 100 U/mL) and human TGF-β (at 20 ng/mL), both from Peprotech (NJ, USA). At days 1 and 3, AA (Sigma-Aldrich, MI, USA) was added at a final concentration of 200 µM, and at day 6 cells were harvested for phenotype analysis. All cell cultures were maintained at 37°C and 5% CO_2_.

### Adoptive Transfer Experiments

To evaluate *in vivo* the direct effect of VitC on naive CD4^+^ T cells, these were isolated from a Foxp3/GFP reporter mouse spleen and sorted based on CD4^+^CD62L^hi^CD44^lo^Foxp3/GFP^−^ phenotype, and intravenously (i.v.) injected into RAG-KO mice a day before skin surgery (4 × 10^5^ cells per mouse). To test the effect of VitC on both natural and iTreg, conventional CD4^+^ Foxp3/GFP^−^ T cells were sorted from a Ly5.1 Foxp3/GFP reporter mouse, and nTreg were obtained from a Ly5.2 Foxp3/GFP mouse using a mouse CD4^+^ CD25^+^ Regulatory T Cell isolation kit (MACS Miltenyi, Germany). A day before surgery, RAG-KO recipient mice received intravenously 1:1 mixture of CD4^+^ conventional T cells and Treg. Grafting was performed as previously described and AA (Sigma-Aldrich, MI, USA) was delivered daily in the water at 0.86 mg/mL from the day of surgery until the end of the experiment.

### *In Vitro* and *Ex Vivo* Suppression Assays

For *in vitro* assay, CD4^+^ Treg cells were sorted from the spleen of a Ly5.1 Foxp3/GFP reporter mouse, as described above, and incubated with VitC (at 50 µM) for 1 h before coculture with antigen presenting cells (APCs) and CD4 conventional T cells. 1 × 10^5^ mitomycin C-treated APCs (FACS sorted from the spleen of a Ly5.2 Foxp3/GFP mouse, based on CD4^−^ CD8^−^ MHC-II^+^ phenotype), 5 × 10^4^ CFSE-labeled CD4^+^ conventional T cells and Tregs (at 1:1, 1:2, and 1:4 Treg:CD4^+^ T effector ratio) were cultured in 96 round well-bottom plates with complete RPMI medium, in the presence of soluble anti-CD3 (clone 2c11, 5 µg/mL) for 3 days. Mitomycin C was used at 50 µg/mL and CFSE at 5 µM (Molecular Probes, Invitrogen, USA). For *ex vivo* experiments, Tregs were isolated at day 20 from draining lymph nodes (DLNs) of RAG^−/−^ transplanted mice, treated or not with VitC, using a mouse CD4^+^ CD25^+^ Regulatory T Cell isolation kit (MACS Miltenyi, Miltenyi). The assay was set up as described above. Cell division was analyzed by flow cytometry to determine responder T cell proliferation (by CFSE dilution) and FlowJo used to determine division index of responder cells. Suppression was calculated with the formula% Suppression = (1 − DI_Treg_/DI_Tresp_) ×100% (where DI_Treg_ stands for the division index of responder cells with Tregs, and DI_Tresp_ stands for the division index of responder cells activated without Tregs).

### Flow Cytometry

Cell samples were stained with anti-CD4 (clone RM4-5), anti-CD8 (clone 53-6.7), anti-CD44 (clone IM7), anti-CD62L (clone MEL-14), anti-CD19 (clone 6D5), anti-CD25 (clone PC61), anti-Foxp3 (clone FJK-16s), anti-CD45.1 (clone A20), anti-CD45.2 (clone 104), and anti-CD11c (clone N418) (all from BioLegend, CA, USA, except Foxp3 from EBioscience, San Diego, CA, USA). All antibodies were conjugated with FITC, phycoerythrin, peridinin chlorophyll protein, or allophycocyanin. All flow cytometry was performed using a BD FACS Canto II cytometer (BD Biosciences, CA, USA), and data were analyzed using FlowJo software (Tree Star, OH, USA).

### ELISA Assay

Supernatants from Treg induction assays were collected at day 6 and stored at 80°C until cytokine quantification by sandwich ELISA. Briefly, 96 well flat-bottomed plates were coated overnight with anti-IFNγ, anti-IL-17, or anti-IL-10 capture antibodies at a final concentration of 1 µg/mL. After several washing steps with PBS 1× + 0.05% Tween 20 and a blocking step with PBS 1× + 10% FBS, samples and standard curve were incubated at room temperature for 2 h. Biotinylated antibodies in conjunction with HRP–avidin were used for detecting the immobilized cytokine and tetramethylbenzidine (TMB, ThermoFisher) substrate solution was added to detect HRP activity. The reaction was stopped by adding sulfuric acid 0.16 M and absorbance measured at 450 nm using a Tecan absorbance microplate reader.

### Quantitative Real-time PCR (qRT-PCR)

RNA from spleen/DLNs sorted-populations or *in vitro* cultured cells were treated for RNA isolation using TRIzol reagent (ThermoFisher, NH, USA), according to manufacture instructions. cDNA was prepared using iScript cDNA synthesis kit (Bio-Rad, CA, USA) and the expression of SVCT2 and housekeeping gene 18S was measured by real-time PCR, using HOT FIREPol EvaGreen qPCR Supermix (Solis BioDyne, Tartu, Estonia) with Mx3000P quantitative PCR system (Agilent Technologies, CA, USA). Primer sequences are showed in Table 1.

**Table 1:** Primer sequences used for conventional and real time PCR analysis.

Gene	Forward (5′–3′)	Reverse (5′–3′)
18S	GCCCGAAGCGTTTACTTTGA	TTGCGCCGGTCCAAGAATTT
SVCT2	CCTGTGTATATTTCTGGGGC	GCCAAAAATGCAAAAGCACT
IL-17	AGCAGCGATCATCCCTCAAA	CTTCATTGCGGTGGAGAGTCC
IL-10	TGGGTTGCCAAGCCTTATCG	AGAAATCGATGACAGCGCCTC
TGF-β	CTGCTGACCCCCACTGATAC	GGGCTGATCCCGTTGATTTC
IFN-γ	GCCAAGTTTGAGGTCAACAACC	ATCTCTTCCCCACCCCGAAT
CD73	GCGGGGAAGTACCCATTCAT	TGATGGTCGCATCTTCAGGA
CD39	CTGAGAGGAAGACCAAGAGGC	ATCCCATACTTAACATTTTCTGGCA
Granzyme B	ATGCTGCTAAAGCTGAAGAGT	TTCCCCAACCAGCCACATAG
Perforin	TTGGTGGGACTTCAGCTTTCC	CCATACACCTGGCACGAACT

### VitC Measurement in Plasma Samples

The 500 µl of blood was collected by cardiac puncture from naive and skin transplanted C57BL/6 mice in tubes coated with heparin. Samples were centrifuged at 3,000 rpm for 15 min and plasma collected and stored at −80°C until analysis. VitC measurements were performed using the methylene blue assay under acidic conditions described by Dilgin et al. (41).

### Statistical Analysis

Statistical significance was determined by Student’s *t*-test, using GraphPad Prism (GraphPad Software, CA, USA).

## Results

### Tregs Express Higher Levels of VitC Transporter

**Figure 1:**
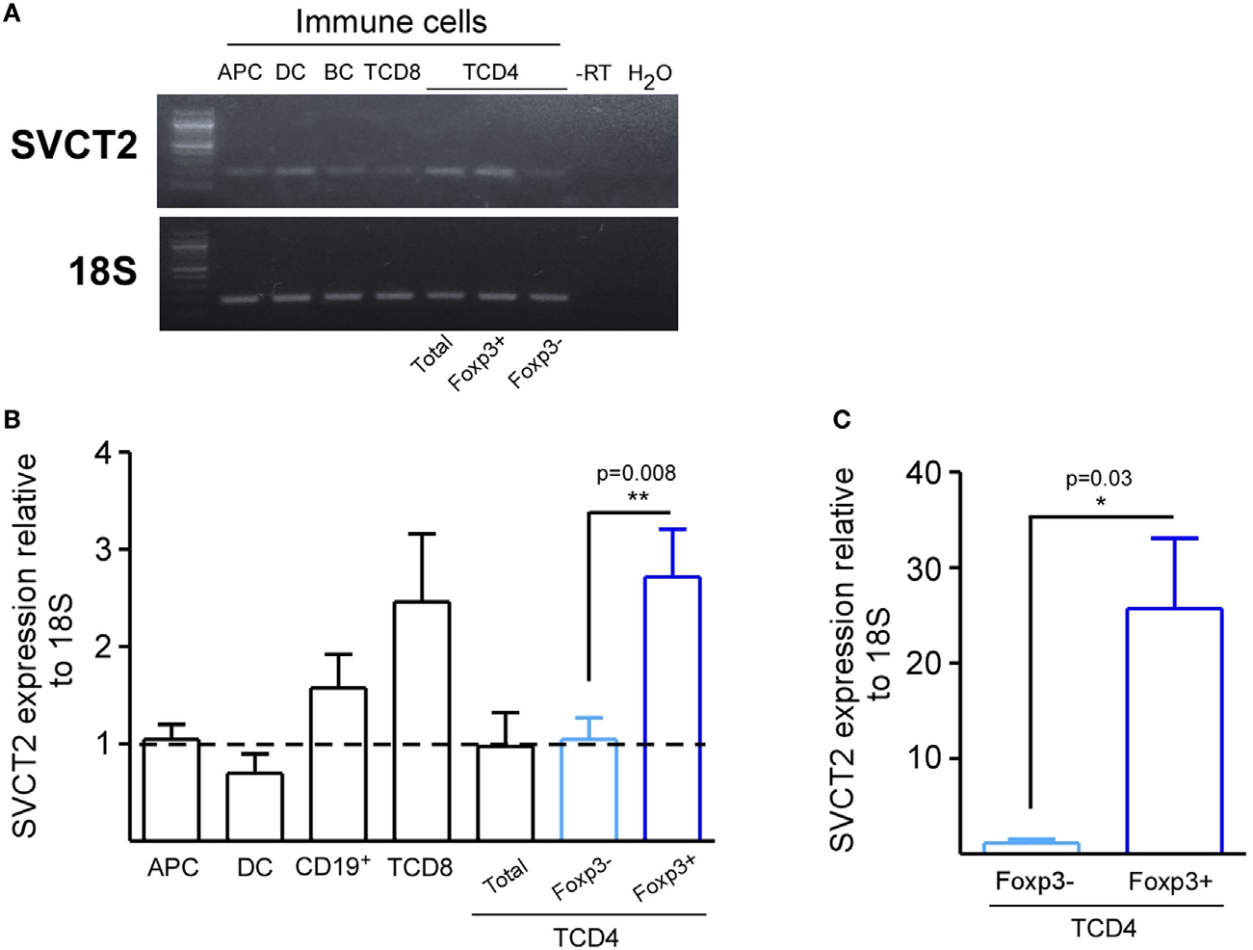
CD4^+^ Foxp3/GFP^+^ regulatory T cells express the highest levels of SVCT2 expression among other leukocyte populations. Antigen presenting cells (APCs), dendritic cells, B cells, CD8^+^ T cells, total CD4^+^ T cells, CD4^+^ Foxp3/GFP^+^ [regulatory T cell (Treg)], and CD4^+^ Foxp3/GFP^−^ T cells (Tconv) were isolated from the spleens of naive Foxp3/GFP reporter mice. **(A)** Real-time PCR (RT-PCR) for SVCT2 and housekeeping gene 18S in isolated immune cells. **(B,C)** Quantification of SVCT2 expression relative to 18S, by RT-PCR in freshly isolated immune cells **(B)**, and sorted Tregs and Tconv after 3 days of polyclonal activation **(C)**. Data represent mean ± SD (*n* = 5 of two independent experiments in B and *n* = 3 of three independent experiments in C). Parametric statistical analysis with unpaired Student’s *t*-test was performed. *P < 0.05, **P < 0.005, ***P < 0.001.

Due to the existence of few studies considering VitC as a potential mediator of immune tolerance, we sought to evaluate SVCT2 on different leukocyte subsets. For this purpose, we FACS sorted total splenic APCs (CD3^−^MHC-II^+^), conventional DCs (CD11c^+^ CD11b^+^), B cells (CD19^+^ CD3^−^), total CD8^+^ and CD4^+^ T cells, conventional CD4^+^ T cells (CD4^+^ Foxp3/GFP^−^), and Tregs (CD4^+^ Foxp3/GFP^+^), and evaluated SVCT2 expression by qRT-PCR. SVCT2 mRNA was detected in all cell subsets (Figure 1A), but Tregs and CD8^+^ T cells showed the highest levels in comparison with total CD4^+^ T cells, ~2.5-fold increase (Figure 1B). Interestingly, SVCT2 mRNA levels were dramatically upregulated on Tregs upon *in vitro* polyclonal activation (~25-fold increase, compared with CD4^+^ Foxp3/GFP^−^ T cells) (Figure 1C). Thus, Tregs correspond to one of the main leukocyte subsets expressing SVCT2, suggesting the ability to sense VitC, which may be higher under T cell activation conditions.

### VitC-Treated nTregs Fail to Suppress Effector CD4^+^ T Cell Proliferation

**Figure 2:**
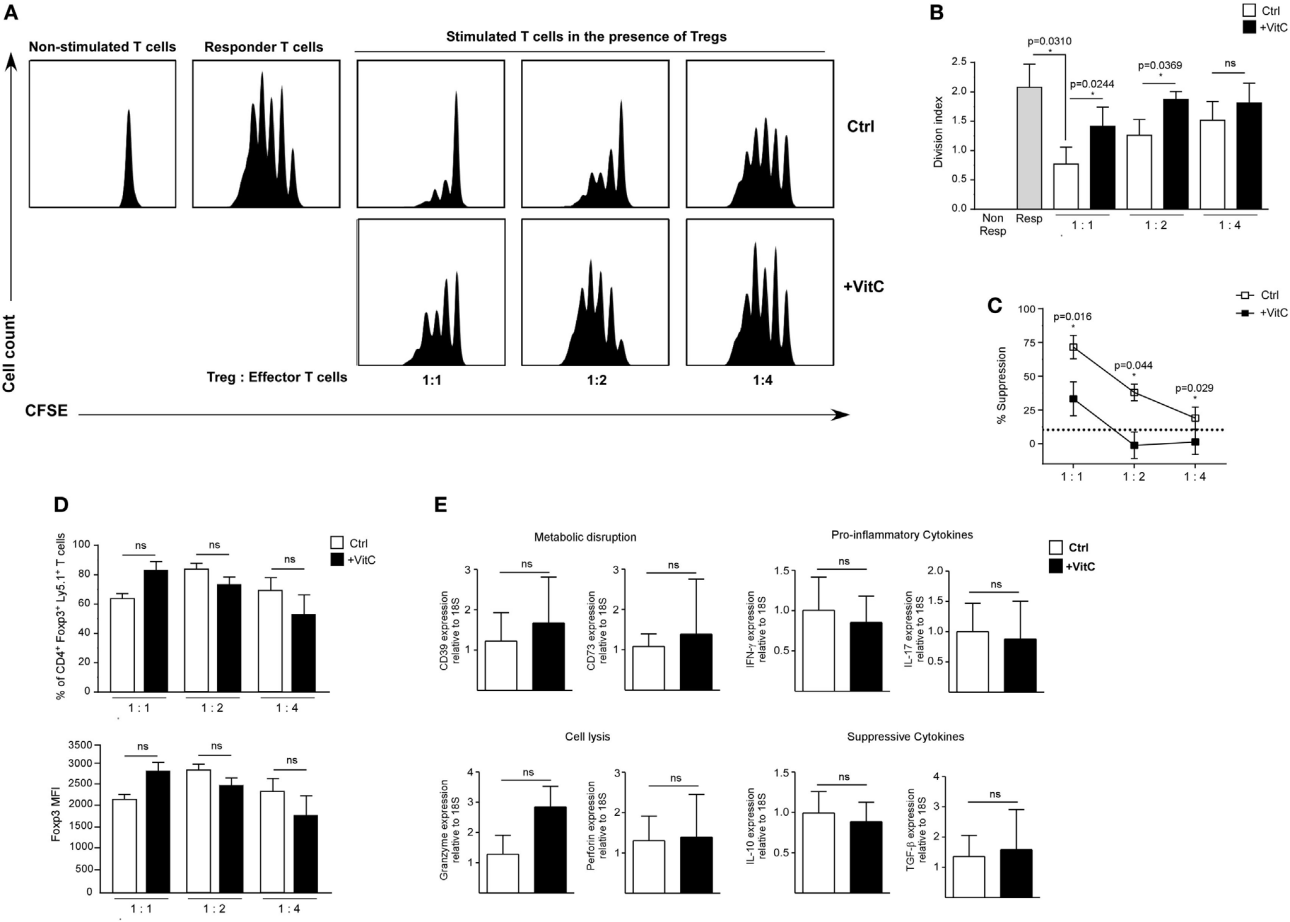
Vitamin C (VitC)-treated natural regulatory T cells (nTregs) are unable to inhibit *in vitro* CD4^+^ T cell proliferation. Analysis of VitC effect on nTreg suppressive function *in vitro*. **(A)** Proliferation curves of non-stimulated CD4^+^ T cells, responder T cells and stimulated CD4^+^ T cells in the presence of nTreg pretreated or not with VitC. **(B)** Quantification of proliferation index (calculated as described in Section “Materials and Methods”) in responder T cells alone (gray bar), CD4^+^ T cells in the presence of control nTreg (white bars) or VitC-treated nTreg (black bars). Division index represent mean ± SD (*n* = 8 for each group of three independent experiments). **(C)** Quantification of percent of suppression in CD4^+^ T cells cocultured with control nTreg (white dots) or VitC-treated nTreg (black dots). Data represent mean ± SEM (*n* = 8 for each group of three independent experiments). **(D)** Percentage of Foxp3^+^ Ly5.1^+^ CD4^+^ T cells (nTregs), and Foxp3 expression level (measured by MFI) in the *in vitro* suppression assay with control (white bars) or VitC-treated (black bars) Treg. Data represent mean ± SD (*n* = 8 for each condition of three independent experiments). **(E)** Expression analysis by qRT-PCR of proinflammatory cytokines (IFN-γ and IL-17), anti-inflammatory cytokines involved in suppression functions (IL-10 and TGF-β), key molecules involved in effector T cell lysis (perforin and granzyme), and ectonucleotidases involved in IDO generation (CD39 and CD73) in *in vitro* activated nTreg. Parametric statistical analysis with paired Student’s *t*-test was performed. *P < 0.05.

Next, we tested VitC influence on the suppression capacity of nTreg cells. For this purpose, we isolated splenic nTregs from a Foxp3/GFP reporter mouse, and pretreated them with VitC before coculture with CFSE-labeled effector CD4^+^ T cells and APCs, FACS-sorted from the spleen of a Foxp3/GFP reporter mouse. After three days of polyclonal activation with soluble anti-CD3, we measured CFSE dilution on effector CD4^+^ T cells by flow cytometry. Our results indicate that VitC pretreated Tregs were less suppressive than non-treated Tregs, detecting lower number of divisions of effector CD4^+^ T cells in the latter (Figures 2A,B). Decreased function, which can also can be expressed in terms of percentage of suppression, was observed when 1:1, 1:2, and 1:4 Tregs:effector CD4^+^ T cells ratios were tested (Figure 2C). In addition, no significant changes on Foxp3^+^ Tregs frequency nor Foxp3 expression levels were observed (Figure 2D), suggesting that the poor suppressive capacity observed on VitC pretreated nTregs was not due to the loss of Foxp3 expression.

In order to define the potential mechanism targeted by VitC in Tregs, we evaluated gene expression by qRT-PCR of pro-inflammatory and Tregs signature cytokines aside from other relevant proteins. In these experiments, we did not detect major changes in the expression of genes involved in metabolic disruption (CD39 and CD73), lytic activity (Granzyme and Perforin), proinflammatory cytokines (IFN-γ and IL-17), or suppressive cytokines (IL-10 and TGF-β) (Figure 2E). Altogether, our findings indicate that VitC affects Tregs function without impacting Foxp3 expression (or other major Treg-associated genes), cell proliferation, or survival.

### VitC Enhances Treg Generation *In Vitro and In Vivo* with No Beneficial Impact on Skin Transplant Survival

**Figure 3:**
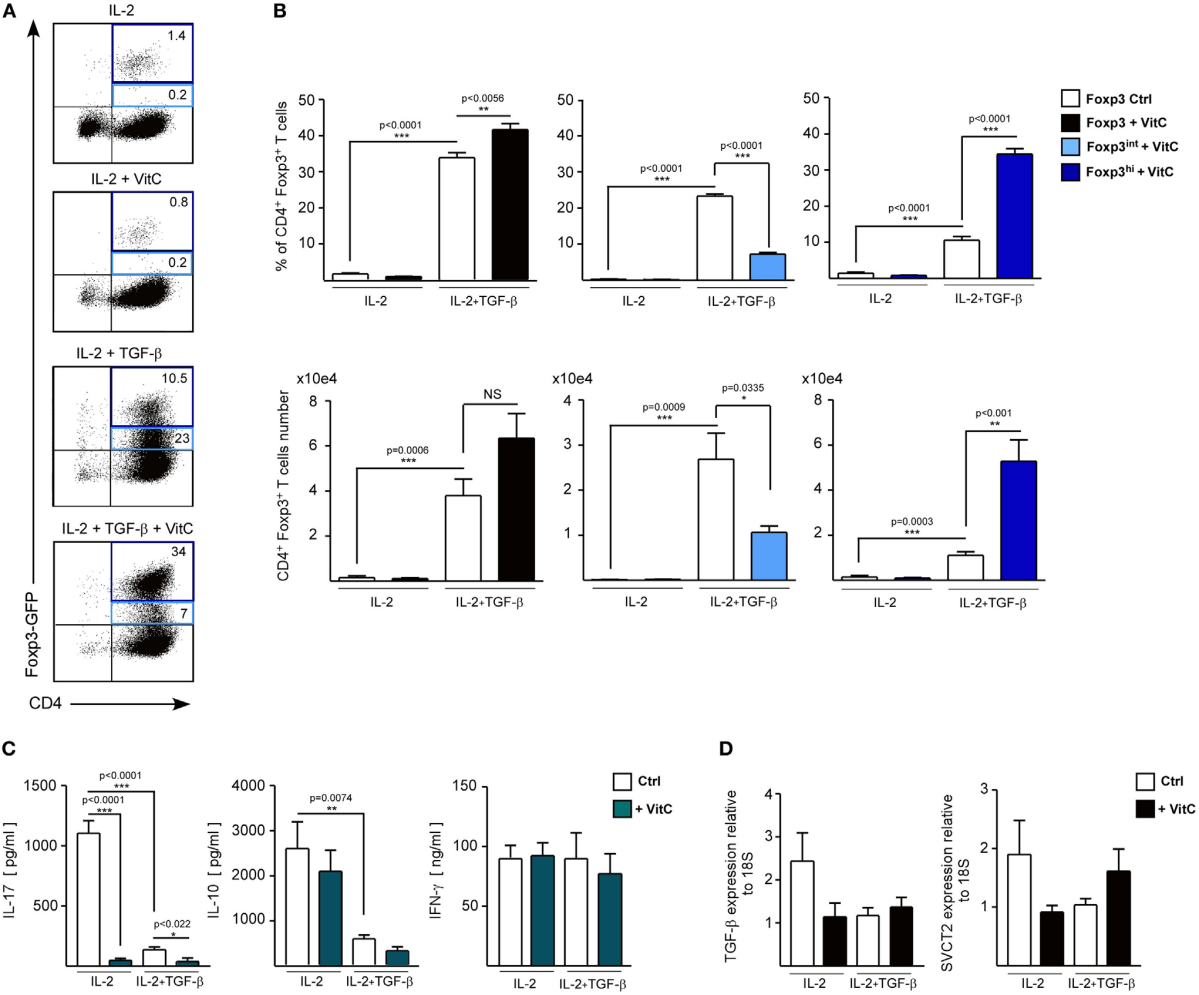
Vitamin C (VitC) triggers the conversion of CD4^+^ Foxp3^−^ T cells toward CD4^+^ Foxp3^+^ T cells *in vitro*. Total splenic CD4^+^ T cells were purified from Foxp3/GFP reporter mice and cultured in the presence of IL-2 and TGF-β for 6 days. At days 1 and 3, VitC was added at a final concentration of 200 µM. **(A)** Representative flow cytometry analysis of induced regulatory T cell (iTreg) induction with or without TGF-β and VitC. **(B)** Quantification of frequency and cell number of total CD4^+^ Foxp3/GFP^+^ T cells, CD4^+^ Foxp3/GFP^int^, and CD4^+^ Foxp3/GFP^hi^ Treg cells, with or without TGF-β and VitC. **(C)** Supernatants of iTreg cultures were analyzed for presence of IL-17, IL-10, and IFN-γ (by ELISA). **(D)** Expression analysis for TGF-β and SVCT2 in iTreg generated with or without VitC. Results represent mean ± SD (*n* = 6 for each group of two independent experiments). Parametric statistical analysis with paired Student’s *t*-test was performed. **P < 0.005, ***P < 0.001.

Recent studies have shown that VitC supplementation during induction of *in vitro* Tregs with TGF-β increases Foxp3 expression (35, 36). In addition, it has been shown that VitC promotes demethylation of the conserved CpG-rich noncoding sequence 2, a regulatory region within the first intron of the Foxp3 gene that is crucial for maintaining stable expression of Foxp3 (35, 36). This increased Foxp3 stability has also been detected after *in vitro* restimulation and after *in vivo* transfer of these cells in unchallenged mice (36). However, it is not known whether VitC could induce the differentiation of Tregs *in vivo*, or whether these *in vivo*-generated iTregs are functional. To answer these questions, we recapitulated Treg induction *in vitro* experiments in the presence of VitC, which demonstrated that total CD4^+^ T cells cultured in the presence of IL-2 and VitC do not upregulate Foxp3 expression (~0.9% of CD4^+^ Foxp/GFP^+^ T cells versus ~1.9% in IL-2 alone) (Figure 3A, top plot). As expected, ~35% of total CD4^+^ T cells cultured with IL-2 and TGF-β differentiated toward iTregs, but the inclusion of VitC enhanced iTregs conversion to ~45% and their number (Figure 3A, last two dot plots and 3B, top left column graph). Furthermore, we observed that iTregs differentiated without VitC contain a population of Tregs with intermediate levels of Foxp3 (Foxp3^int^), representing ~70% of total Tregs (Figure 3B). Interestingly, the inclusion of VitC in iTregs differentiation conditions shifted this Foxp3^int^ population toward a population with higher levels of Foxp3 expression (Foxp3^hi^), increasing from 10.6 ± 2.5% in control cultures to 34.4 ± 3.3% (Figure 3B). Furthermore, these VitC-iTregs are able to suppress *in vitro*, in the absence of retinoic acid (RA), as previously reported (36), and as shown in Figure S1D in Supplementary Material, in which the % of proliferating cells is depicted when cocultured with nTreg, iTreg generated in the absence of VitC (iTreg control) and iTreg generated in the presence of VitC (iTreg^+^ VitC); each condition at 1:1, 1:2, and 1:4 proliferating:Treg cell ratios. More clearly, Figure S1E in Supplementary Material displays the % of suppression under the mentioned culture conditions (~50–80% for nTreg, ~75–80% for iTreg control, and >85% for iTreg^+^ VitC). The same data set demonstrates the inversely correlated frequencies of both effector T (A) and Treg (B) cells under the coculture conditions, and the unchanged Foxp3 expression on Treg cells in the cultures (C). Furthermore, Figure S3 in Supplementary Material displays the post-sorting purity of naïve CD4^+^ T cells used in the previously described in vitro assay, and Figure S4 in Supplementary Material demonstrates that only signal from proliferating effector CD4^+^ T cells is detected, excluding potential staining of proliferating Treg cells. In addition, cytokine expression and secretion was evaluated using quantitative RT-PCR and ELISA, respectively. As observed in Figure 3C, IL-17 production was negatively regulated by VitC in both control (IL-2) and iTregs (IL-2 and TGF-β) conditions (from 1104 ± 255 pg/mL in control Tregs versus 58 ± 35 pg/mL in VitC-treated Tregs with IL-2; and from 137 ± 54 pg/mL in control Tregs versus 36 ± 74 pg/mL in VitC-treated Tregs with IL-2 and TGF-β). Additionally, VitC affected neither IL-10 nor IFN-γ secretion. The same findings were obtained when TGF-β and SVCT2 expression were analyzed by qRT-PCR (Figure 3D). Thus, VitC may inhibit IL-17 secretion from *in vitro* activated CD4^+^ T cells, favoring a milieu for iTreg differentiation and/or stabilization.

**Figure 4:**
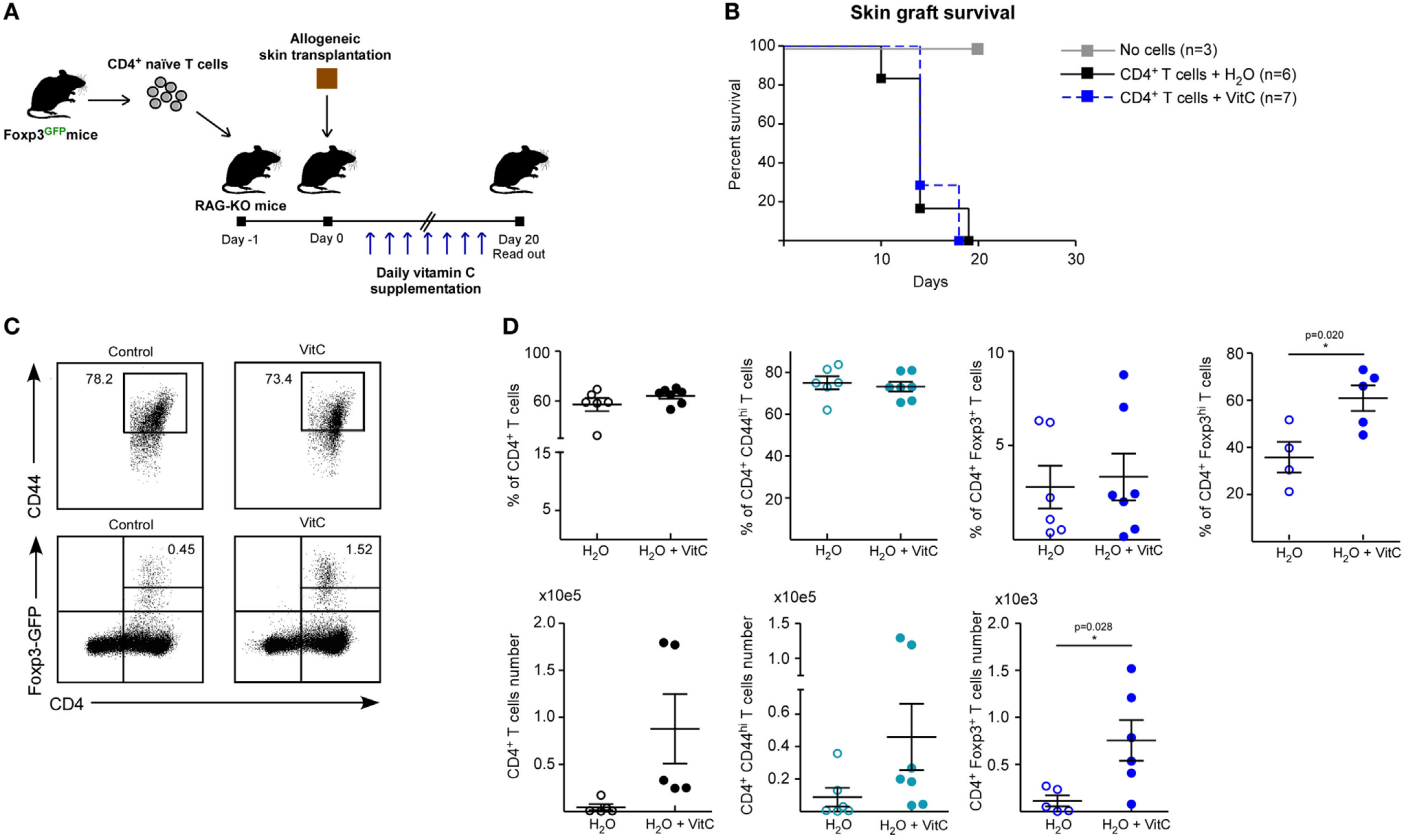
Vitamin C (VitC) allows for the conversion of CD4^+^ Foxp3/GFP^−^ T cells to CD4^+^ Foxp3/GFP^+^ T cells in skin transplanted RAG-KO mice. **(A)** Schematic representation of the experimental design. C57BL/6 RAG-KO mice received a single intravenous (i.v.) injection of naive CD4^+^CD62L^hi^CD44^lo^Foxp3/GFP^−^ T cells from Foxp3/GFP reporter mice a day before allogeneic skin transplantation. Daily VitC supply in the water (0.86 mg/mL) was maintained for 20 days after surgery. **(B)** Kaplan–Meier plots showing skin graft survival. **(C)** Representative flow cytometry plots of regulatory T cell (Treg) analysis from draining lymph nodes (DLNs) of transplanted mice. **(D)** Quantification of total CD4^+^ T cells, activated CD4^+^ CD25^+^ CD44^+^ T cells, and CD4^+^ Foxp3/GFP^+^ Treg cells frequency and number in DLNs. Results are mean ± SD (*n* = 6 for each group of two independent experiments). Parametric statistical analysis with unpaired Student’s *t*-test was performed. **P < 0.005, ***P < 0.001.

Next, we assessed the effect of VitC on both Treg generation and function *in vivo* using a previously standardized model to study immune tolerance based on allogeneic skin transplantation in RAG-KO mice, receiving adoptive transfer of naive CD4^+^ Foxp3/GFP^−^ T cells the day before surgery (6). For *in vivo* VitC supplementation, skin transplanted mice received a daily mega-dose of VitC (0.86 mg/mL) in the drinking water, for 20 days (Figure 4A), which was corroborated by measuring VitC levels in their serum (Figure S2 in Supplementary Material). During this time, skin transplants were monitored for signs of rejection, observing that VitC-supplemented mice reject at the same kinetic than control mice (Figure 4B). Analysis of CD4^+^ T cells from skin transplant-DLNs showed no changes in the frequencies of total CD4^+^ T cells, CD4^+^ Foxp3^−^CD44^hi^ effector T cells nor total CD4^+^ Foxp3/GFP^+^ Tregs (Figures 4C,D). However, similar to what we observed during *in vitro* iTregs differentiation, a population of iTregs with high expression of Foxp3 was significantly increased in mice treated with VitC, representing ~60% of total Tregs (Figure 4D). In terms of cellularity, VitC significantly increased the number of total iTregs (Figure 4D, bottom graphs). These results show that VitC also induces the differentiation of iTregs *in vivo*; however, this increase does not translate into graft tolerance, suggesting that VitC may impact Tregs subsets, effector CD4^+^ T cells or both.

### VitC Increases Treg Generation *In Vivo* with No Beneficial Impact on Skin Transplant Survival

**Figure 5:**
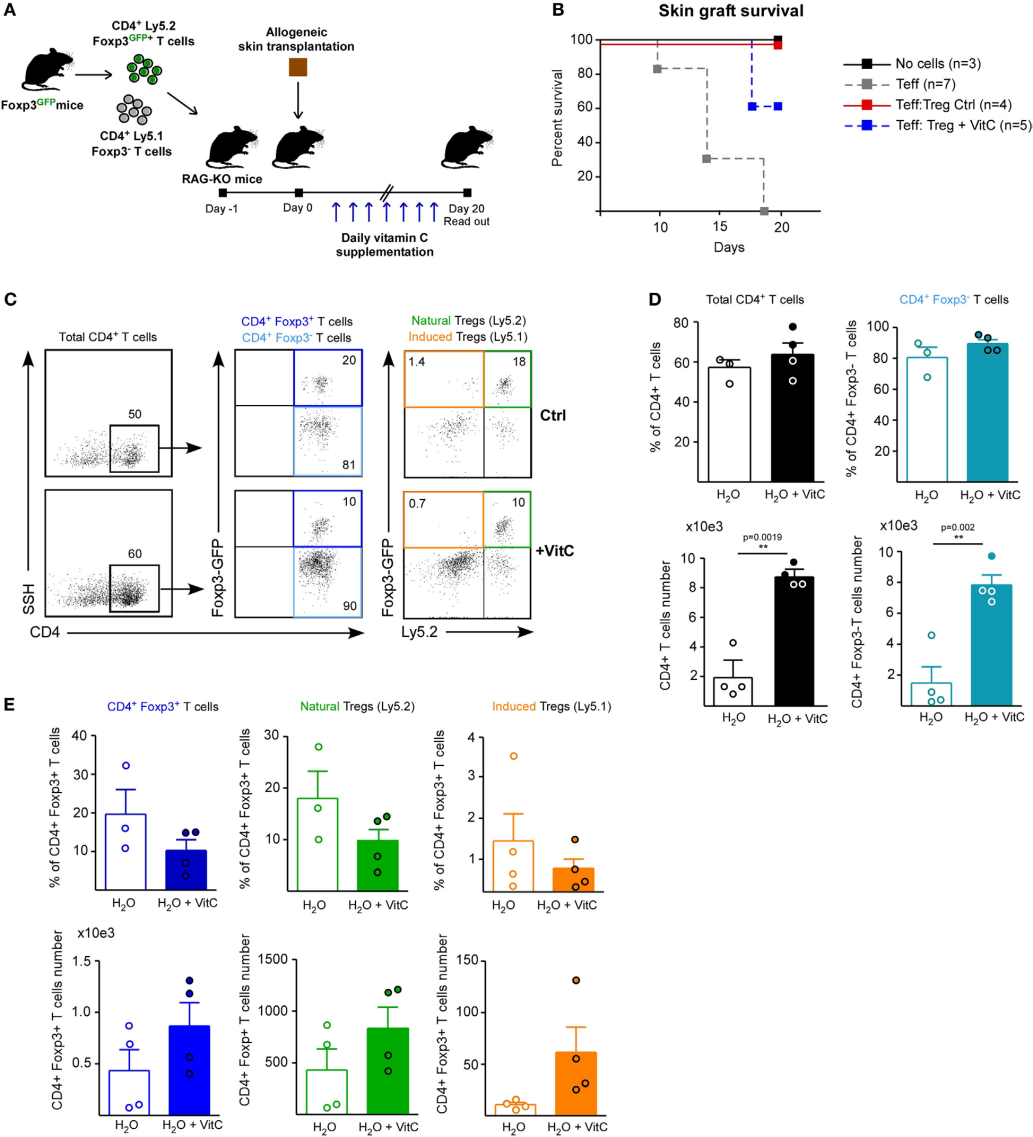
Vitamin C (VitC) increases both natural regulatory T cell (nTreg) and induced Treg (iTreg) *in vivo* using a skin transplantation model. **(A)** Schematic representation of the experimental design. C57BL/6 RAG-KO mice received a single i.v. injection of a mixture of CD4^+^ Foxp3/GFP^−^ and CD4^+^ Foxp3/GFP^+^ T cells from Foxp3/GFP reporter mice a day before allogeneic skin transplantation. Daily VitC supply in the water (0.86 mg/mL) was maintained until the end of the experiment. At day 20, draining lymph nodes (DLNs) were collected. **(B)** Kaplan–Meier plots showing skin graft survival. **(C)** Flow cytometry analysis of nTreg and iTreg based on Foxp3/GFP and Ly5.1/Ly5.2 congenic expression. **(D)** Quantification of total CD4^+^ T cells and effector T cells frequency and number in DLNs. **(E)** Quantification of total, nTreg, and iTreg frequency and number in DLNs. Results are mean ± SD (*n* = 4 of two independent experiments). Parametric statistical analysis with unpaired Student’s *t*-test was performed. **P < 0.005, ***P < 0.001.

In order to define whether VitC treatment can also expand the nTreg population and alter their function *in vivo*, we performed adoptive transfer of a mixture of T cells a day before skin transplantation: Treg from a Ly5.2^+^ Foxp3/GFP^+^ mouse and conventional CD4^+^ Foxp3/GFP^−^ T cells from a Ly5.1^+^ Foxp3/GFP mouse (Figure 5A). As before, VitC was added daily into drinking water and DLNs were analyzed by flow cytometry at day 20 postsurgery. Kaplan–Meier plots show, as expected, that grafted mice without cell transfer do not reject (Figure 5B, black line), while mice receiving only effector CD4^+^ T cells start rejection after two weeks post-surgery (Figure 5B, gray line). As it has been shown in other studies, adoptive cotransfer of Treg allows for graft acceptance (Figure 5B, red line), however, some of the mice treated with VitC rejected the graft despite receiving Treg (Figure 5B, blue line).

Discriminating by congenic marker and Foxp3/GFP expression, we studied total CD4^+^ T cells, nTregs (Foxp3/GFP^+^ Ly5.2^+^) and iTregs (Foxp3/GFP^+^ Ly5.1^+^) in response to VitC (Figure 5C), finding that VitC did not alter effector T cell frequency, but exhibit a tendency to decrease the frequency of total, nTreg and iTreg, by 50% (Figures 5D,E). However, when cell number was determined, we observed a tendency to augment the number of all Treg cell populations (with no significant difference), but effector CD4^+^ T cells were enriched in response to VitC supplementation (Figures 5D,E), suggesting that VitC may promote effector T cell expansion (Figure 5D).

### VitC Negatively Affects Treg Suppression Capacity *Ex Vivo*

**Figure 6:**
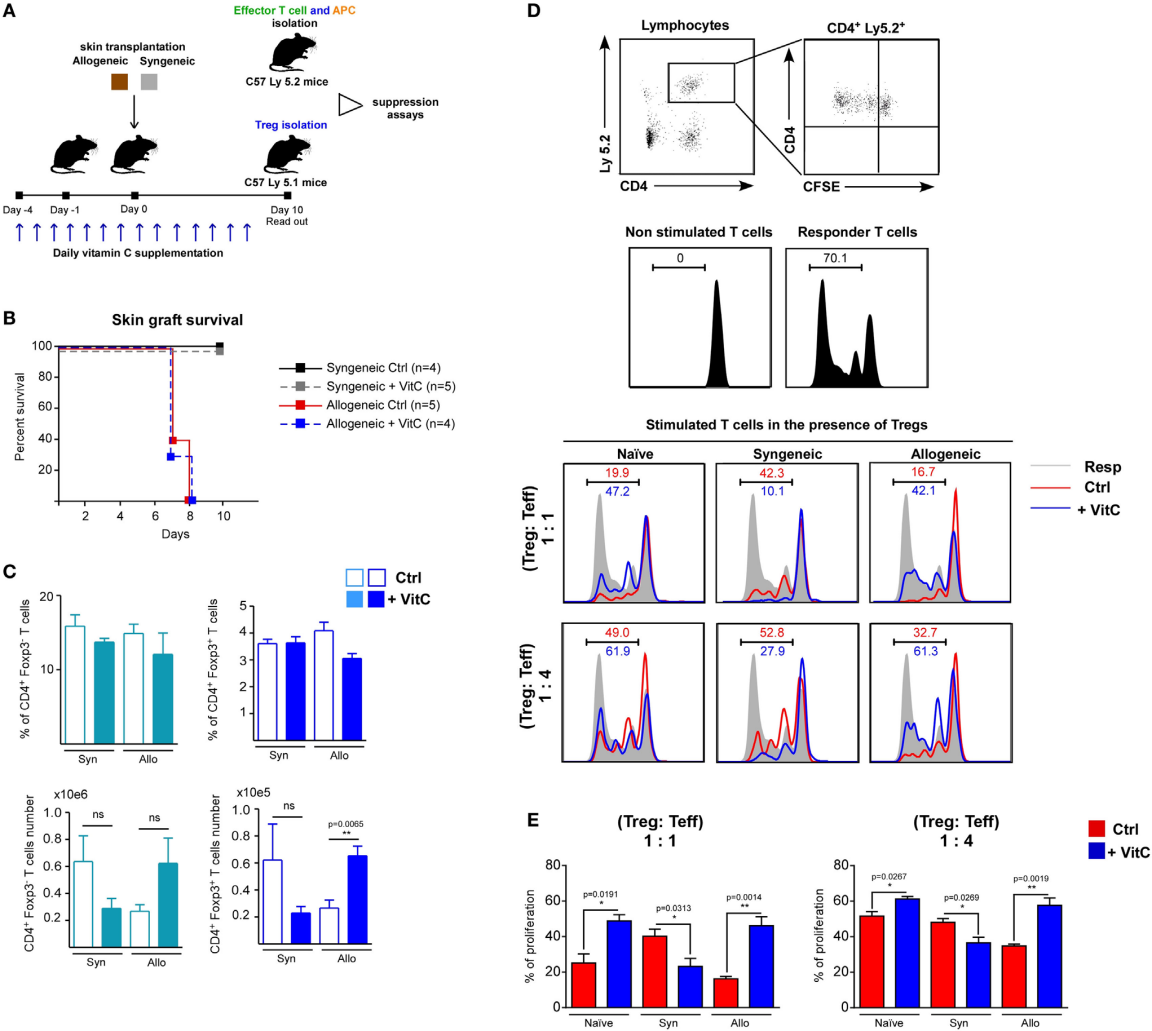
Vitamin C (VitC) enriches for regulatory T (Treg) cells with defective suppressive function in the skin-transplanted mice. **(A)** Schematic representation of the experimental design. Syngeneic and allogeneic skin transplantation was performed on C57BL-6 wild-type mice receiving VitC in water 4 days prior to surgery until the end of the experiment. After 10 days of skin transplantation, Tregs were isolated for suppression assay. **(B)** Kaplan–Meier plots showing skin graft survival. **(C)** Quantification of effector CD4^+^ T cells and Treg frequency and cell number in draining lymph nodes (DLNs) from control mice (white bars) and VitC-treated mice (colored bars). Results are mean ± SD (*n* = 4 of one independent experiment). **(D)** Gating strategy for analyzing CFSE dilution profile of non-stimulated effector CD4^+^ T cells alone or CD4^+^ T cells cocultured with antigen presenting cells (APCs) (shade) in the presence of Treg isolated from control (red line) and VitC-treated (blue line) transplanted animals. **(E)** Quantification of effector CD4^+^ T cells proliferation as percentage of T cells with low levels of CFSE. Results are mean ± SD (*n* = 4 of one independent experiment). Parametric statistical analysis with unpaired Student’s *t*-test was performed. **P < 0.005, ***P < 0.001.

Regardless of VitC augmenting the cell number of CD4^+^ T cell populations, and favoring iTreg differentiation, its impact on *in vivo* Tregs functionality appears unaltered. In order to corroborate this, we performed syngeneic and allogeneic skin transplants on wild-type C57/BL6 mice (receiving or not VitC in drinking water), and then isolated sufficient number of Treg to further test their suppressive activity *ex vivo* (Figure 6A). First, we administered VitC in water the same day mice were transplanted (day 0) obtaining no changes on Treg cells (data not shown); then, we decided to deliver VitC in water from day 4 aiming at “priming” the immune system before the surgery. Graft survival analysis showed that syngeneic transplanted mice (both control, black line, and VitC-treated, gray line) do not reject their graft, while allogeneic transplanted mice show rejection signs between 7 and 8 days of surgery, without detecting differences between control, red line and VitC-treated mice, blue line (Figure 6B). After 10 days postsurgery, DLNs quantification analysis showed no changes in the frequencies of CD4^+^ Foxp3/GFP^−^ CD44^hi^ activated T cells or total CD4^+^ Foxp3/GFP^+^ Treg in VitC-treated mice compared with non-treated controls (Figure 6C, top graphs). As in our previous results, VitC treatment increases the number of CD4^+^ Foxp3/GFP^+^ Treg in the allogeneic group (from ~20,000 Tregs in untreated mice to ~60,000 Tregs in VitC-treated mice), as displayed in Figure 6C. *Ex vivo* suppression assays using Treg isolated from dLN of naive, syngeneic and allogeneic mice (obtained from controls and VitC-treated animals) confirmed that VitC does not improve Treg-mediated suppression, on the contrary, Treg isolated from VitC-treated naive and allogeneic mice are less suppressive than controls (Figures 6D,E). This decreased suppression capacity was also detected when a lower ratio of Treg:effector T cells (1:4) was used in the assay (Figures 6D,E). Intriguingly, Treg cells isolated from VitC-treated syngeneic animals were more suppressive than untreated animals, at both Treg:T effector cell ratios tested.

Overall, our work indicates that Treg express the SVCT2 transporter and increase the level of Foxp3 expression in response to VitC. Despite our *in vitro* results, *in vivo* VitC supplementation enriches for Treg, but this does not translate into allograft acceptance. Even more, when VitC-treated Treg are tested for suppressive function *ex vivo*, their regulatory activity was indeed decreased.

Even though recent literature involving VitC as an enhancer of *in vitro*-differentiated Treg is promising, our data highlight a differential behavior of *in vivo*-expanded Treg in a VitC supplemented state.

## Discussion

Regulatory T cells are a key subpopulation of T lymphocytes that contributes to maintain the immunological homeostasis by suppressing effector T cells proliferation and function. Because of their clinical relevance in different pathological settings, such as autoimmune diseases (e.g., lupus, ulcerative colitis, rheumatoid arthritis, among others) and solid organ transplant rejection, different studies have tested the ability of small molecules to enhance the generation, expansion and function of Treg cells (42, 43). Current protocols for clinical application of human Treg infusion include the isolation and *in vitro* expansion of the patient’s nTregs using anti-CD3/CD28-coated dynabeads and IL-2 with or without rapamycin (43, 44). On the other hand, in spite of several murine studies successfully achieving a protocol to improve the induction of Treg cells from naive CD4^+^ T cells, using TGF-β, RA, and/or rapamycin (26, 45, 46), its translation to human Treg has not been effective enough, as shown in studies in which human Treg generated *in vitro* fail to retain Foxp3 expression and suppress graft versus-host disease (47). For these reasons, the search for new protocols is urgent and a better characterization of the phenotype and functionality of these iTreg is warranted.

Recent reports have shown that VitC potentiates the generation of iTreg *in vitro*, by increasing the expression levels of the master regulator gene Foxp3 through an epigenetic mechanism (35–37). Moreover, DNA methylation status of additional Treg signature genes, such as CTLA-4 and Eos, has been shown to actively demethylate during the induction of Treg in the presence of VitC (37). These VitC induced-Treg cells also favor allogeneic skin transplantation tolerance when adoptively transferred into RAG-KO mice, which have been featured as a promising finding for clinical application (37).

Despite this new data, however, the effect of VitC on endogenous nTreg has been neglected thus far; an important matter considering that both types of Treg are known to be distinct in terms of phenotype and function (48), and that most clinical trials use either umbilical cord blood- or peripheral blood-derived nTreg (49, 50). Furthermore, the *in vitro* and *in vivo* function of VitC-treated Treg has not yet been studied, considering that Nikolouli’s report only describes the role of VitC on TGF-β^+^ RA-iTreg differentiated in the presence of allogeneic APCs (without declaring a potential effect of both vitamins on the APCs or the secretion of vitamins from APCs). Taking the above into consideration, we aimed to characterize the effect of VitC on nTreg and iTreg cells *in vitro* and *in vivo* using a mouse model for skin transplantation.

We began our study by determining the expression of VitC transporter SVCT2, which until now has not been studied in detail, although SVCT2 expression has been reported in human macrophages (39), murine T cells (29), and human T cells (40). Interestingly, our qRT-PCR data show that among the leukocyte populations analyzed, Treg display the highest levels of SVCT2 expression, which is even enhanced upon activation, implying that Treg could be more susceptible to VitC incorporation and thereby, to its modulatory effect on Treg functionality and stability.

Driven by the recent literature on Treg induction in the presence of VitC, we expected a beneficial effect of VitC treatment on nTreg cells; however, our results show that nTreg cells pretreated with VitC before coculturing with effector CD4^+^ T cells fail to suppress T cell proliferation, which has not been described before. Since Treg cells suppression capability is mediated by several distinctive mechanisms [reviewed by Sakaguchi et al. (4)], we evaluated the expression of key suppressive genes to determine which of them might be compromised upon VitC treatment; thereby accounting for the decreased Treg functionality. Neither amino acid metabolism-related (CD39 and CD72), cytotoxicity-related (perforin and Granzyme B), nor cytokines-related genes (IL-10, TGF-β, IFN-γ, nor IL-17) were modulated by VitC, discarding the possibility of Treg acquiring a proinflammatory phenotype or adopting impaired production of lytic granules or inhibitory cytokines. The lack of changes in gene expression agrees with RNA-seq data of *in vitro* VitC-treated iTreg obtained from Nikolouli et al. (37), and might suggest other types of suppression mechanisms have been targeted by VitC such as microvesicles production or regulation of the metabolic state of Treg (51, 52), which we did not include in the current report. Although, there is no evidence of VitC regulating such processes, its ability to drive a metabolic switch in other cell types has been reported (53), leaving open the possibility of exploring this concept in T cells.

When we used VitC to differentiate Tregs from naive CD4^+^ T cells *in vitro*, same as in other studies (35–37), we observed that VitC treatment enhanced the generation of suppressive CD4^+^ Foxp3/GFP^+^ T cells (Figure 3 and Figure S1 in Supplementary Material), but interestingly, we detected a shift from a population of iTreg with intermediate levels of Foxp3 toward a population with higher levels of Foxp3 expression, an observation not reported until now. This CD4^+^ Foxp3/GFP^int^ T cell population could correspond to a transient Foxp3 expression in non-Treg cells, which has been reported in other studies (54, 55), meaning that VitC abrogates this process, favoring Treg generation with high levels of Foxp3. Accordingly, IL-17 levels were decreased with VitC treatment, independent of TGF-β presence, which could indicate that VitC is able to inhibit Th17 conversion in the presence of IL-2. Interestingly, a recent report shows that VitC could upregulate the expression of IL-17 under Th17 polarizing conditions in the absence of IL-2 signaling, which seems to depend on jmjd2 histone demethylase activity (56), showing that VitC may tune the immune response by targeting T helper cell differentiation. This statement is also supported by reports in which other vitamins, such as vitamin A, modulate the Th17/Treg axis depending on the concentration of vitamin A used (57).

The results from our *in vivo* experiments revealed that VitC treatment also allows for the differentiation of iTreg. However, our *in vivo* experienced VitC-Treg were unable to drive transplantation tolerance. Failing to facilitate skin graft survival could be related to a deficiency in skin (graft) homing or decreased suppression capacity, as shown in *ex vivo* suppressive assay (Figure 6). Furthermore, we cannot dismiss a putative VitC effect on conventional CD4^+^ Foxp3/GFP^−^ T cells, as we also observed an increase in the number of these effector T cells, in addition to augmented IFN-γ production when activated in the presence of VitC. An unfavorable effect of VitC on the activity of Treg populations in combination with VitC influence on effector T cell subsets, could partially explain the overall result obtained using this *in vivo* approximation.

The RAG-KO mouse model is a widely used approach in immunology because of its usefulness in dissecting the contribution of specific T (or B) cell populations under different experimental settings, but based on its special genetic background, any extrapolation of the observations made in this model toward a more clinically relevant one must be made with caution. For this reason, we also included an evaluation of VitC treatment in immune competent mice. Similar to what we observed in RAG-KO mice, VitC supplementation increased both effector and Treg cell number, with no beneficial impact on skin graft acceptance. Most importantly, Treg isolated from VitC-treated animals showed decreased suppression capacity *ex vivo*, corroborating our previous *in vitro* and *in vivo* data.

We acknowledge that VitC supplementation through water consumption has some drawbacks, such as our inability to accurately measure the amount of water each mouse actually ingested, which could account for the variability obtained in our data during the performance of *in vivo* experiments. Moreover, VitC is recognized for not being chemically stable in aqueous solution for long periods of time, due to its oxidization to dehydroascorbic acid, which led us to replace VitC-supplemented water on a daily basis. However, the rationale behind our administration route for VitC delivery was to emulate regular administration in humans in a non-invasive way. Considering the potential pitfalls of our protocol, we were able to detect an increase in VitC plasmatic levels in treated mice (Figure S2 in Supplementary Material).

Altogether our results show that VitC treatment acts in a distinct fashion on nTreg and iTreg, both *in vitro* and *in vivo*, without improving Treg function. On the other hand, VitC could act on effector CD4^+^ T cells as well, which could complement the mechanistic behind the rejection of the skin transplants observed in this study. Lastly, we believe that the nutritional status of patients, particularly regarding plasmatic vitamins levels, is an important parameter to take into consideration, as accumulating evidence shows the role of vitamins in tuning the immune response.

## Ethics Statement

This study was carried out after the revision and approval of its experimental protocol in accordance with the recommendations of bioethical Committee guidelines from the Faculty of Medicine, Universidad de los Andes, and the Comisión Nacional de Ciencia y Tecnología, CONICYT.

## Author Contributions

KO performed experiments, analyzed data, and wrote the manuscript. MC-M performed experiments. TG-C performed experiments. KP-L designed experiments, analyzed data, and wrote the manuscript.

## Conflict of Interest Statement

The authors declare that the research was conducted in the absence of any commercial or financial relationships that could be construed as a potential conflict of interest.

## Supplementary Material

Figure S1 Vitamin C (VitC)-induced regulatory T cells (Tregs) suppressive function *in vitro*. **(A)** Frequency of responders Ly5.2^+^ CD4^+^ T cells after 3 days of activation in the presence of natural Treg (nTreg) or induced Treg (iTreg). **(B)** Frequency of Ly5.2^−^ CD4^+^ Treg cells after 3 days of coculture with responder T cells. **(C)** Geometric median intensity of fluorescence (gMFI) of Foxp3 on Foxp3/GFP^+^ Ly5.2^−^ CD4^+^ T cells in the suppression assay. **(D)** Frequency of proliferative responder T cells, measured by CFSE dilution and Division Index (DI) determination, as described in Section “Materials and Methods.” **(E)** Suppressive function of Treg measured by *in vitro* suppression percentage, as described in Section “Materials and Methods.” Data represent mean ± SD (*n* = at least 5 for each group of three independent experiments). Parametric statistical analysis with paired Student’s *t*-test was used in **(D)** (****P* < 0.001 versus responder cells; ^##^*P* < 0.01 versus nTreg; ^#^*P* < 0.05 versus nTreg; ^&&&^*P* < 0.001 versus control iTreg; ^&&^*P* < 0.01 versus control iTreg). Two-way ANOVA was used in **(E)** (***P* < 0.01 VitC-iTreg versus Ctrl-iTreg; ****P* < 0.001 VitC-iTreg versus Ctrl-iTreg; ^#^*P* < 0.05 Ctrl-iTreg versus nTreg; ^###^*P* < 0.001 Ctrl-iTreg versus nTreg).

Figure S2 Daily vitamin C (VitC) oral supplementation increases plasmatic levels of VitC in unchallenged and syngeneic transplanted mice but not allogeneic transplanted mice. **(A)** Absorbance curve for VitC detection through methylene-blue method. **(B)** Quantification of plasmatic VitC concentration through methylene-blue method in samples obtained from naive, syngeneic, and allogeneic skin transplanted C57BL/6 wild-type mice after 10 days of receiving daily VitC supply in drinking water.

Figure S3 Sorting strategy and purity confirmation of FACS sorted naive CD4^+^ CD25^−^ CD44^−^ T cells for adoptive transfer experiments. Total CD4^+^ T cells were enriched from Foxp3/GFP mice with a CD4^+^ T cell isolation kit and stained for CD4, CD62L, and CD44 for FACS sorting of a CD4^+^Foxp3/GFP^−^CD62L^hi^CD44^lo^ population.

Figure S4 “Gating strategy for analyzing proliferation of responder cells in suppression assays.” CD4^+^ effector T cells were purified from Ly5.1 Foxp3/GFP reporter mice spleens to use as responder cells in our suppression assays. The flow cytometry plots confirms their purity, showing lack of Foxp3 expression post-sort, with no significant Foxp3 expression after several days of culture in the absence of TGF-β. After 3 days of coculture with APC and Tregs derived from Ly5.2 Foxp3/GFP mice, proliferation of responder cells was analyzed based on CFSE dilution, gated on CD4^+^ Ly5.1^+^ T cells. Flow cytometry plots shows only effector T cells (Teff) exhibit a dilution pattern in the CFSE channel, proving this gating strategy allow us to measure proliferation with CFSE without interference of GFP signals.
