# Supplementary figures and images for: Vitamin C Fosters the In Vivo Differentiation of Peripheral CD4+ Foxp3− T Cells into CD4+ Foxp3+ Regulatory T Cells but Impairs Their Ability to Prolong Skin Allograft Survival
Source: Front Immunol. 2018 Feb 9;9:112. doi: 10.3389/fimmu.2018.00112 (PMC5811461; doi:10.3389/fimmu.2018.00112)

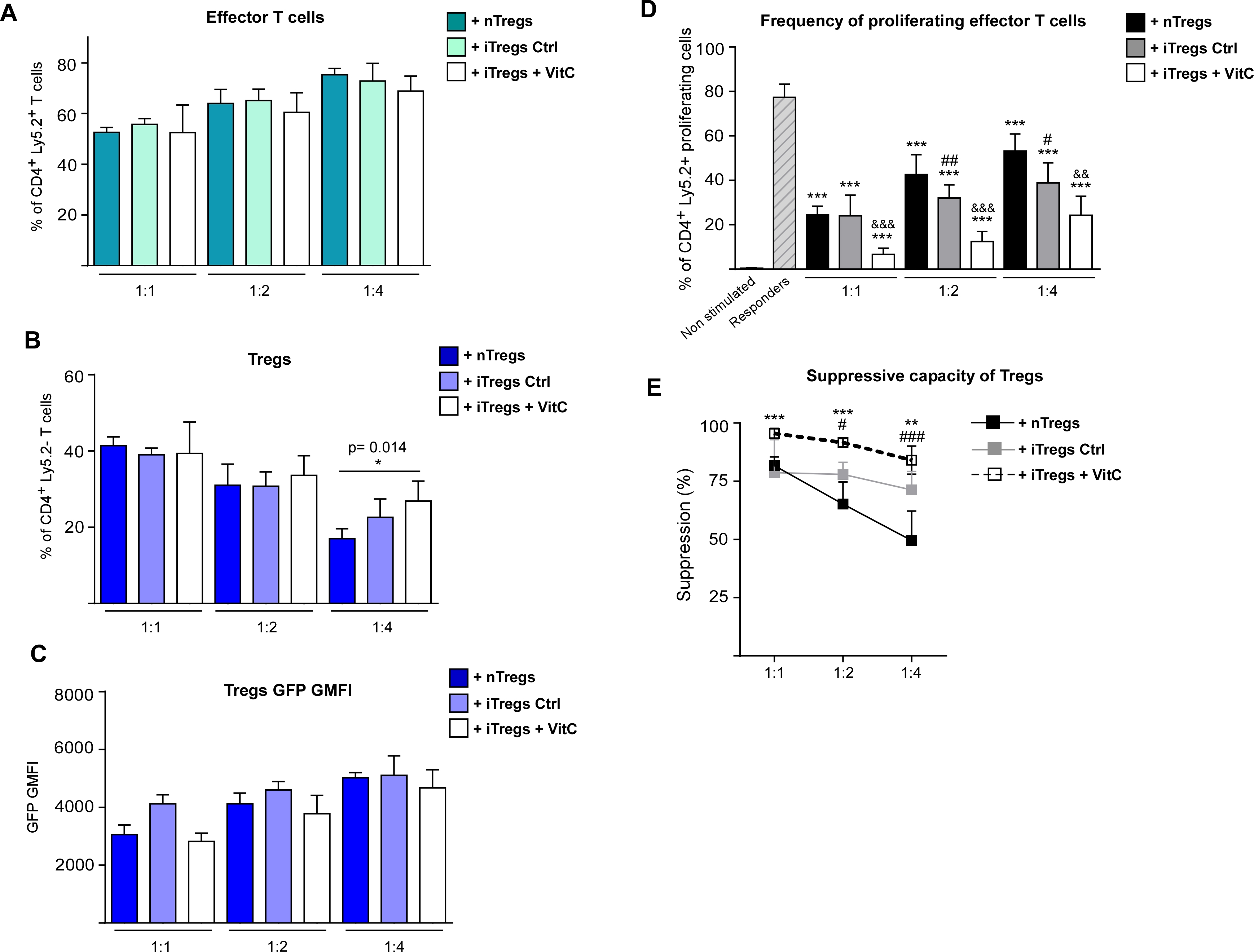

Supplement: Figure S1 — Vitamin C (VitC)-induced regulatory T cells (Tregs) suppressive function in vitro. (A) Frequency of responders Ly5.2+ CD4+ T cells after 3 days of activation in the presence of natural Treg (nTreg) or induced Treg (iTreg). (B) Frequency of Ly5.2− CD4+ Treg cells after 3 days of coculture with responder T cells. (C) Geometric median intensity of fluorescence (gMFI) of Foxp3 on Foxp3/GFP+ Ly5.2− CD4+ T cells in the suppression assay. (D) Frequency of proliferative responder T cells, measured by CFSE dilution and Division Index (DI) determination, as described in Section “Materials and Methods.” (E) Suppressive function of Treg measured by in vitro suppression percentage, as described in Section “Materials and Methods.” Data represent mean ± SD (n = at least 5 for each group of three independent experiments). Parametric statistical analysis with paired Student’s t-test was used in (D) (***P < 0.001 versus responder cells; ##P < 0.01 versus nTreg; #P < 0.05 versus nTreg; &&&P < 0.001 versus control iTreg; &&P < 0.01 versus control iTreg). Two-way ANOVA was used in (E) (**P < 0.01 VitC-iTreg versus Ctrl-iTreg; ***P < 0.001 VitC-iTreg versus Ctrl-iTreg; #P < 0.05 Ctrl-iTreg versus nTreg; ###P < 0.001 Ctrl-iTreg versus nTreg). [file Image_1.JPEG]

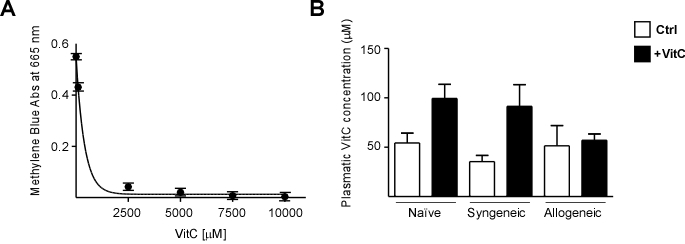

Supplement: Figure S2 — Daily vitamin C (VitC) oral supplementation increases plasmatic levels of VitC in unchallenged and syngeneic transplanted mice but not allogeneic transplanted mice. (A) Absorbance curve for VitC detection through methylene-blue method. (B) Quantification of plasmatic VitC concentration through methylene-blue method in samples obtained from naive, syngeneic, and allogeneic skin transplanted C57BL/6 wild-type mice after 10 days of receiving daily VitC supply in drinking water. [file Image_2.JPEG]

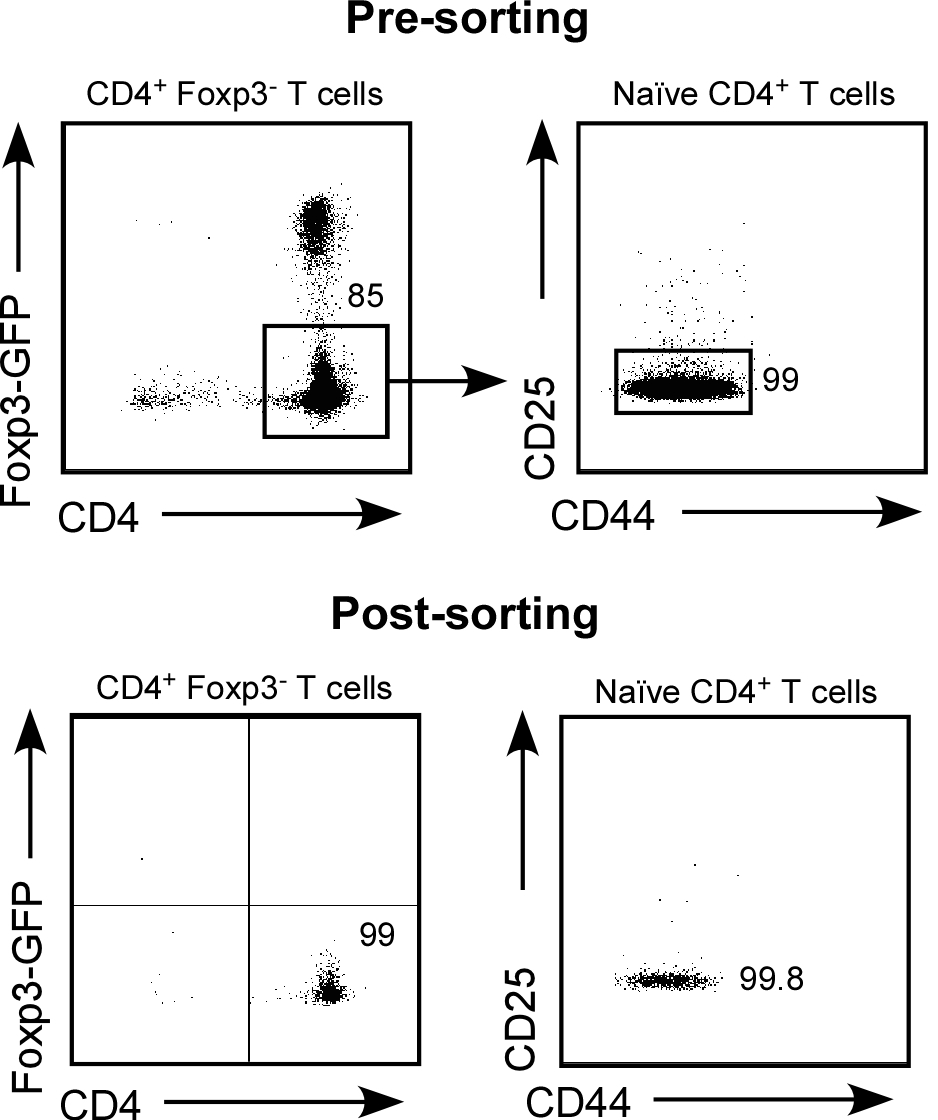

Supplement: Figure S3 — Sorting strategy and purity confirmation of FACS sorted naive CD4+ CD25− CD44− T cells for adoptive transfer experiments. Total CD4+ T cells were enriched from Foxp3/GFP mice with a CD4+ T cell isolation kit and stained for CD4, CD62L, and CD44 for FACS sorting of a CD4+Foxp3/GFP−CD62LhiCD44lo population. [file Image_3.JPEG]

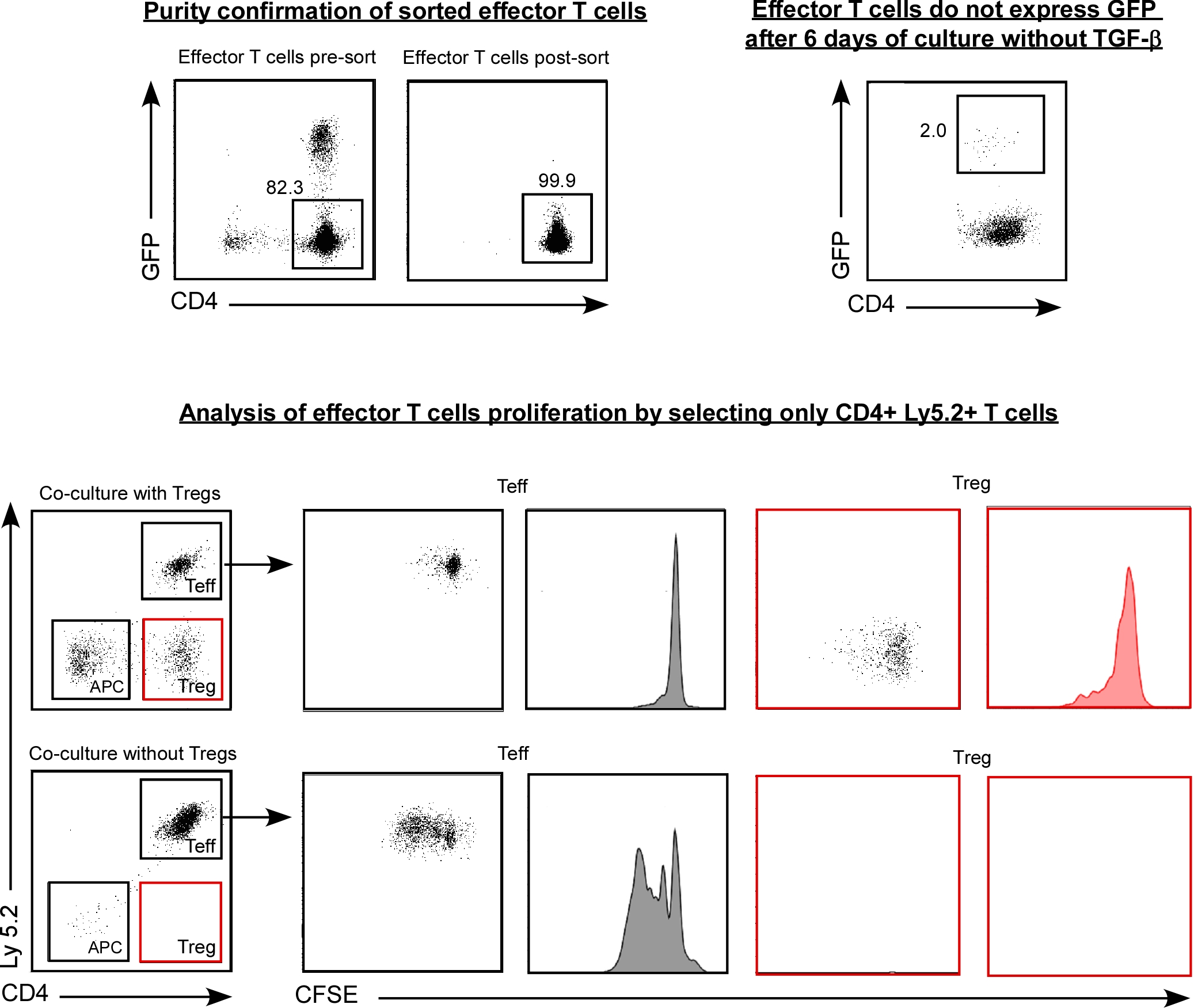

Supplement: Figure S4 — “Gating strategy for analyzing proliferation of responder cells in suppression assays.” CD4+ effector T cells were purified from Ly5.1 Foxp3/GFP reporter mice spleens to use as responder cells in our suppression assays. The flow cytometry plots confirms their purity, showing lack of Foxp3 expression post-sort, with no significant Foxp3 expression after several days of culture in the absence of TGF-β. After 3 days of coculture with APC and Tregs derived from Ly5.2 Foxp3/GFP mice, proliferation of responder cells was analyzed based on CFSE dilution, gated on CD4+ Ly5.1+ T cells. Flow cytometry plots shows only effector T cells (Teff) exhibit a dilution pattern in the CFSE channel, proving this gating strategy allow us to measure proliferation with CFSE without interference of GFP signals. [file Image_4.JPEG]
